# Association Between the Rate of Treatment Response and Short-Term Outcomes in Childhood Guillain-Barré Syndrome

**DOI:** 10.3389/fneur.2021.746389

**Published:** 2021-11-05

**Authors:** Mei Jin, Libo Zhao, Jing Liu, Weijin Geng, Ziwei Zhao, Chunzhen Li, Jingru Xue, Suzhen Sun

**Affiliations:** ^1^Department of Pediatrics, Hebei Medical University, Shijiazhuang, China; ^2^Department of Pediatric Neurology, Children's Hospital of Hebei Province, Shijiazhuang, China

**Keywords:** Guillain-Barré syndrome, children, treatment response rate, Hughes Functional Grading Scale, short-term outcomes

## Abstract

**Introduction:** Few studies have examined the association between the rate of treatment response and the outcome of pediatric Guillain-Barré syndrome (GBS). Therefore, our study aimed to identify treatment response in relation to the short-term outcomes of GBS. Further, we investigated its potential predictive value for prognosis.

**Methods:** Our retrospective study included children diagnosed with GBS in the Pediatric Neurology Department of the Children's Hospital of Hebei Province from 2016 to 2020. According to the rate of response from the standard intravenous immunoglobulin (IVIg) treatment, patients were divided into two groups: rapid-response GBS (initial response within 7 days) and slow-response (initial response within 8–30 days). The GBS disability score (Hughes Functional Grading Scale) was used to assess the children's functional disability at nadir, 1 month, and 6 months after onset.

**Results:** Among the 36 children included in the study, 18 (50%) and 18 (50%) were rapid and slow responders, respectively. Time from IVIg treatment to the initial response was significantly shorter in the rapid-response group (5 [3–6.25] days vs. 10.5[8.75–15] days in slow-response GBS, *p* < 0.001). Hughes score at 1 month was worse than the rapid responders (Fisher's exact test, *p* = 0.006). Survival analysis (Kaplan–Meier) with respect to regaining the ability to walk independently (Hughes Functional Grading Scale of 2) within 1 month after onset was significantly different among the two groups (log-rank test for trend, *p* = 0.024). The abnormal levels of cerebral spinal fluid proteins and autonomic dysfunction were more frequent in the slow-response group than those in the rapid group (*p* < 0.05).

**Conclusion:** The rate of response to IVIg treatment was correlated with short-term outcomes in children with GBS and had predictive value for prognosis. The role of patient's initial responses to treatment could be significantly valuable in developing more effective and efficient treatment options.

## Introduction

Guillain-Barré syndrome (GBS) is an acute polyradiculoneuropathy characterized by progressive, symmetrical upper or lower limb weakness and absent or reduced tendon reflexes, with or without paresthesia (1). Intravenous immunoglobulin (IVIg) is one of the preferred treatments for children with GBS in several centers (2); however, the time from IVIg treatment to the initial response varies. Specifically, some patients have a quick reaction (within 24 h), whereas some patients have a slow response (2 weeks or more, even 30 days). According to previously published reports, the prognosis of most children with GBS was considered good (3). However, some patients did not achieve complete recovery within the first 6 months. Therefore, early identification of poor prognosis in GBS is crucial. Presently, there are several widely used prognostic methods based on data derived from adult patients (4); however, how these scores relate to children remains unknown. In our study, we aimed to identify the association between the rate of treatment response and short-term outcomes of GBS in children. Further, we analyzed the predictive value for prognosis.

## Materials and Methods

### Subjects

We retrospectively recruited patients with GBS (aged <14 years) who were admitted to our hospital between January 2016 and July 2020. Patients who met the US National Institute of Neurological Disorders and Stroke diagnostic criteria (1) and level 1 of the Brighton classification (5), i.e., rapidly progressive symmetrical upper or lower limb weakness with or without sensory signs, absent or decreased deep tendon reflexes, albuminocytological dissociation of cerebrospinal fluid analysis, and nerve conduction slowing and block of electrophysiologic studies, were included in this study. Six, three, two, two, and three patients with acute transverse myelitis, acute flaccid myelitis because of polio or enterovirus, acute-onset chronic inflammatory demyelinating polyradiculoneuropathy, myasthenia gravis, and inflammatory myositis, respectively, were excluded based on these criteria. This study was approved by the Ethics Committee of the Children's Hospital of Hebei Province.

### Methods

In this study, treatment response was defined as an improvement by at least one Hughes Functional Grading Scale after IVIg treatment. The patients were divided into two groups according to the rate of treatment response: rapid-response (initial response within 7 days) and slow-response (initial response within 8–30 days) GBS.

#### Motor Functional Disability Assessment

The Patient's Functional Motor Disability Was Assessed by the GBS Disability Score (Hughes Functional Grading Scale: Grade 0, Normal; Grade 1, Minimal Signs and Symptoms, Able to run; Grade 2, Able to Walk 10 m Unaided, but Unable to run; Grade 3, Able to Walk With aid; Grade 4, bed-Bound and not Able to Lift Legs; Grade 5, Requiring Mechanical Ventilation; And Grade 6, Death) (6) Upon Admission, at Nadir, 1 month, and 6 months After Onset. Patients With Hughes Functional Grade ≥3 at 1 month After Onset Were Classified as Having Poor Outcomes, and Patients With Hughes Functional Grade <3 at 1 month After Onset Were Classified as Having Good Outcomes.

#### Clinical and Laboratory Findings

(1) Cranial nerve involvement, such as facial paralysis and bulbar paralysis; neuropathic pain; autonomic dysfunction (cardiac arrhythmia, hypertension, sweating, and bladder retention); and mechanical ventilation were also analyzed. All patients received a dose of 2 g/kg IVIg treatment (30 patients with a short 2-day course, 6 patients with 5 days) within 24 h of admission. (2) Cerebrospinal fluid (CSF) analysis was performed within 2 weeks of weakness onset. We measured the white blood cell and protein levels. (3) Regarding anti-glycolipid antibodies, we sampled serum and CSF to measure antibodies, such as anti-GM1 and anti-GD1a. (4) For T lymphocyte or thyroid function analysis, we sampled serum to identify immune system function.

#### Electrophysiologic Study

All Patients were applied with serial electrophysiologic study and subclassified into acute inflammatory demyelinating polyneuropathy (AIDP) and acute motor axonal neuropathy (AMAN) according to the Hughes electrodiagnostic criteria (7). The data should include a bilateral or unilateral motor and sensory nerve conduction study, in addition to an F-wave response. The parameters included distal motor latency, motor and sensory conduction velocity, distal and proximal compound muscle action potentials, and minimal F-wave latency. A-waves were identified between the M response and F response. A-waves of the median or ulnar nerves were primarily considered in this study. All patients were divided into A-waves and non-A-waves based on the presence or absence of A-waves.

#### Statistical Analyses

Statistical analyses were performed using the International Business Machines Statistical Package for the Social Sciences Statistics version 24. Categorical data were shown as proportions, and continuous data were shown as the medians with IQR. Differences in proportions were tested by χ^2^tests or Fisher's exact test. The continuous variables were tested by the Wilcoxon rank sum test and Mann–Whitney U analysis. CSF protein levels in the two groups were presented as means with 95% confidence intervals. The Kaplan–Meier analysis with the log-rank test was used to assess the ability to walk independently within 1 month after onset. Statistical significance was set at 0.05.

## Results

### Baseline Clinical Characteristics

**Figure 1:**
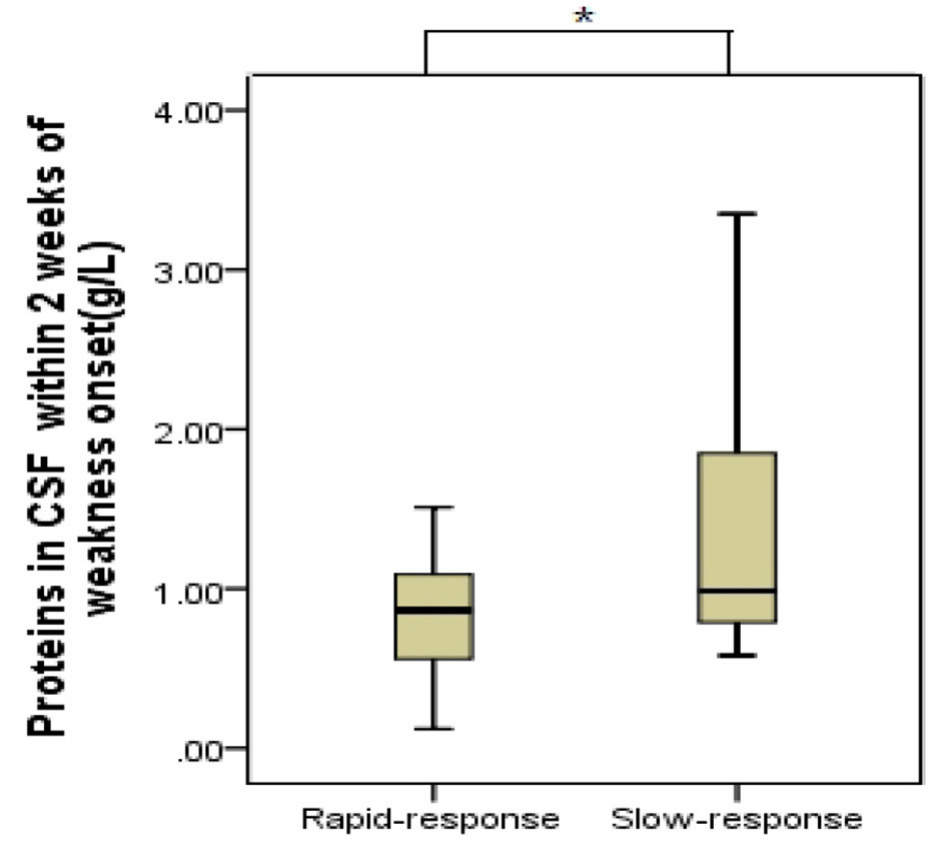
Cerebral spinal fluid (CSF) protein levels in the two groups with Guillain-Barré syndrome. Geometric mean and 95% confidence intervals are shown (box and whiskers: median and 10–90% percentile). Slow-response patients (0.99 g/L) had higher CSF protein levels than the rapid-response (0.87 g/L) (*p < 0.05). Rapid-response: treatment response within 7 days; slow-response: treatment response in 8–30 days.

A total of 36 children with GBS were recruited, with 18 (50%) and 18 (50%) patients having rapid-response GBS and slow-response GBS, respectively. Time from IVIg treatment to the initial response was significantly shorter in the rapid-response group (5 [3–6.25] days vs. 10.5 [8.75–15] days in slow-response GBS, *p* < 0.001). Protein levels in the CSF were presented with albuminocytological dissociation within 14 days of weakness onset. The slow-response group (0.99 g/L, range: 0.78–1.86) had significantly higher CSF protein levels than the rapid-response group (0.87 g/L, range: 0.5–1.09; Wilcoxon rank sum test, *p* < 0.05). Box and whiskers analysis also revealed a difference in the CSF protein level between the groups (Figure 1). Meanwhile, autonomic dysfunction was significantly more frequent in the slow-response group (*p* = 0.015).

No significant differences were noted in terms of sex or age. Furthermore, the differences were not statistically significant between the two groups in terms of cranial nerve abnormality rates, such as facial and bulbar paralysis, and neuropathic pain. Additionally, the group of patients with slow-response GBS, electrophysiological variant (such as AIDP and AMAN), with or without A-waves, laboratory tests (T lymphocyte or thyroid function abnormalities or anti-glycolipid antibody), and duration of hospitalization were also not statistically different. Sociodemographic and clinical data of all the patients are shown in Table 1.

**Table 1:** Sociodemographic and clinical features of patients with childhood GBS.

**Variables**	**Rapid-response (*n* = 18)**	**Slow-response (*n* = 18)**	**Statistic values**	***P-value***
Time from the IVIg treatment to the initial response, days, median (*IQR*)	5 (3–6.25)	10.5 (8.75–15)	*Z* = 5.141	<0.001
Male, n (%)	15 (83.3)	10 (55.6)	χ^2^ = 3.273	0.070
Age, months, median (*IQR*)	66 (48.5–114)	54 (30.75–81)	*Z* = 1.509	0.131
Hughes score on admission, *n* (%)			—	0.113^a^
2	7 (38.9)	3 (16.7)		
3	7 (38.9)	4 (22.2)		
4	4 (22.2)	9 (50)		
5	0	2 (11.1)		
Hughes score at nadir, *n* (%)			—	0.119^a^
2	6 (33.3)	3 (16.7)		
3	7 (38.9)	4 (22.2)		
4	5 (27.8)			
5	0	4 (22.2)		
Hughes score at 1 month after onset, *n* (%)			—	0.006^a^
0	8(44.4)	1 (5.6)		
1	6 (33.3)	2 (11.1)		
2	1 (5.6)	6 (33.3)		
3	3 (16.7)	7 (38.8)		
4	0	1 (5.6)		
5	0	1 (5.6)		
Hughes score at 6 months after onset, *n* (%)			—	0.602^a^
0	15 (83.3)	12 (66.6)		
1	1 (5.6)	3 (16.6)		
2	2 (11.1)	1 (5.6)		
3	0	1 (5.6)		
4	0	1 (5.6)		
Variant, *n* (%)			—	0.658^a^
AIDP	16 (88.9)	14 (77.8)		
AMAN	2 (11.1)	4 (22.2)		
GBS with A-waves, *n* (%)			χ^2^ = 1.87	0.171
Yes	13 (72.2)	9 (50)		
No	5 (27.8)	9 (50)		
Proteins in the CSF, g/L, median (*IQR*)	0.87 (0.5–1.09)	0.99 (0.78–1.86)	*Z* = 1.978	0.048
Neurological symptoms, *n* (%)				
Facial paralysis	2 (11.1)	2 (11.1)	—	1.000^a^
Bulbar paralysis	5 (27.8)	7 (38.9)	χ^2^ = 0.500	0.480
Neuropathic pain	12 (66.7)	10 (55.6)	χ^2^ = 0.468	0.494
Autonomic dysfunction, *n* (%)	3 (16.7)	10 (55.6)	χ^2^ = 5.900	0.015
Laboratory abnormalities, *n* (%)				
T lymphocyte abnormalities	16 (88.9)	14 (77.8)	—	0.658^a^
Thyroid function abnormalities	6 (33.3)	7 (38.9)	χ^2^ = 0.120	0.729^a^
Anti-glycolipid antibody positive	2 (11.1)	4 (22.2)	—	0.658^a^
Treatment, *n* (%)				
IVIg with 2 days	14 (77.8)	16 (88.9)	—	0.658^a^
Plasmapheresis	0	2 (11.1)	—	0.486^a^
Corticosteroids	7 (38.9)	12 (66.7)	χ^2^=2.786	0.095
Mechanical ventilation, *n* (%)	0	4 (22.2)	—	0.104^a^
Duration of hospitalization, days, median (*IQR*)	13.5 (10–22.75)	19.5 (13.75–29.75)	*Z* = 1.919	0.055

*Rapid-response, improvement within 7 days; slow-response, improvement within 8–30 days; IVIg, intravenous immunoglobulin; AIDP, acute inflammatory demyelinating polyneuropathy; AMAN, acute motor axonal neuropathy; GBS, Guillain-Barré syndrome; SD, standard deviation; ^a^Fisher's exact test; Z, Rank sum test; NA, not applicable*.

### Association Between the Rate of Treatment Response and Walking Capacity

**Figure 2:**
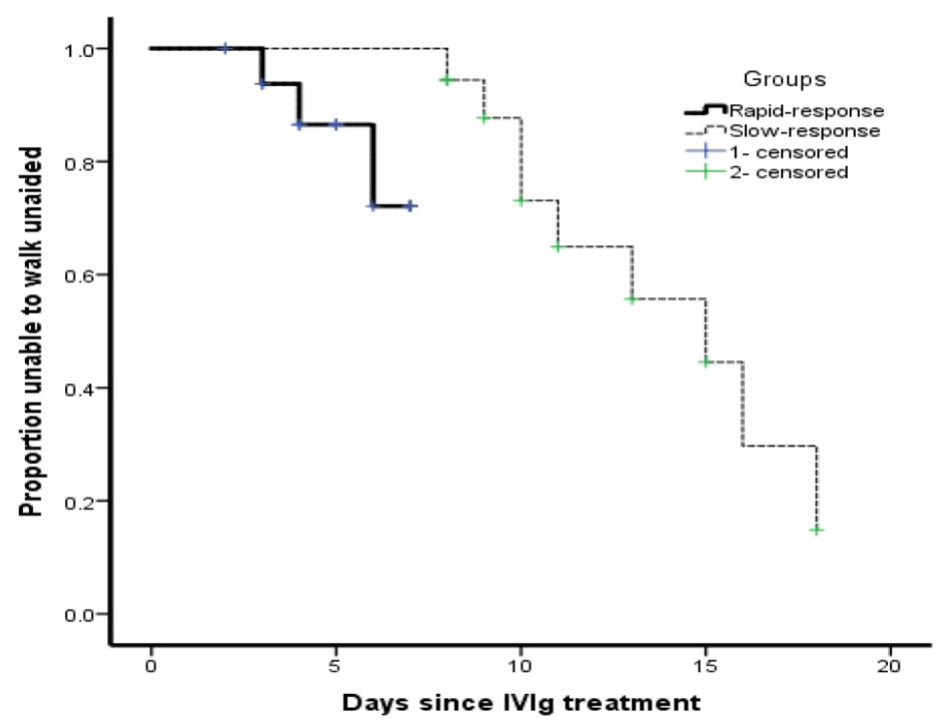
Clinical recovery of patients with Guillain-Barré syndrome in relation to the rate of treatment response within 1 month after onset. The Kaplan–Meier analysis of patients with Guillain-Barré syndrome in children regaining the ability to walk unaided (Hughes Functional Grading Scale of 2) in relation to the different response rates after initiating intravenous immunoglobulin treatment within 1 month after onset.

No significant differences were noted in the Hughes score upon admission, at nadir, and at 6 months after onset between the two groups. However, the Hughes score of the slow responders at 1 month after onset was worse than that of the rapid responders (Fisher's exact test, *p* = 0.006). To assess the potential predictive value of the rate of treatment response for disease outcome, patients were grouped according to the differences in the rate of response after the start of IVIg treatment. Survival analysis (Kaplan–Meier) with respect to regaining the ability to walk unaided (Hughes Functional Grading Scale of 2) within 1 month was significantly different among the two groups (log-rank test for trends, *p* = 0.024, Figure 2).

## Discussion

GBS is presently the most common cause of acute flaccid paralysis in children and is characterized by several clinically distinctive variants and outcomes. The prognosis of GBS was considered good if there was a complete recovery during the first 6 months of treatment, whereas up to 20% of patients remained unable to walk independently, and approximately 7% of patients died (8). In this study, 9 (25%) and 27 (75%) out of 36 patients had poor and good prognoses, respectively, at 1 month after weakness onset, whereas at 6 months after weakness onset, only 2 (5.6%) patients were unable to walk unaided, suggesting that the long-term prognosis of GBS in children was better than that in adults. Prediction models are needed for the early identification of poor outcomes in patients with GBS, who are eligible for additional effective treatment to prevent irreversible nerve degeneration. Currently, there are several widely used prognostic methods, including modified Erasmus GBS outcome score, Erasmus GBS respiratory insufficiency score, increased IgG levels after IVIg treatment, and the risk of respiratory failure (9, 10). However, these prediction methods were derived from adult patients, and the value of these methods in children remains unknown. Therefore, we developed a simple and convenient clinical method to predict patient prognosis. According to the rate of response since IVIg treatment, patients were divided into the rapid-response (within 7 days) group and slow-response group (within 8–30 days). The Hughes score in the slow-response group at 1 month after onset was worse than that of the rapid-response group. To assess the potential predictive value of the rate of treatment response for GBS outcome, survival analysis (Kaplan–Meier) was performed in our study. Survival analysis with respect to regaining the ability to walk unaided (Hughes score ≤ 2) at 1 month after onset was significantly different between the two groups (*p* < 0.05), which suggested that the time of treatment response in children might be defined as 7 days, patients who were beyond 7 days were highly unable to walk unaided and had a poor short-term prognosis.

Laboratory examinations, such as CSF examination and antiganglioside antibody level, could further support the diagnosis of GBS. The classic CSF finding regarding albuminocytological dissociation has been previously described (11). In this study, the slow-response group had a higher CSF protein level than the rapid-response group (*p* < 0.05). Furthermore, Box and whiskers analysis revealed a difference in CSF protein levels in these two groups. This phenomenon could be explained that elevated CSF protein levels mainly come from the damage of peripheral nervous roots (12), and higher protein levels could be related to more serious demyelination and axonal loss, signifying greater disability; therefore, patients showed a slow response to treatment and a worse prognosis.

GBS is a common postinfectious and antibody-mediated autoimmune system disorder. To date, several antiganglioside antibodies have been found (13); however, most of the patients with GBS are still antibody negative. In this study, the antibody positivity rate was relatively low (16.7%); therefore, more efforts are needed to explore the mechanisms of unknown antibodies in GBS. Meanwhile, consistent with our study, a high incidence rate (83.3%) of T lymphocytes indicated that T lymphocytes were involved in the pathogenesis of GBS, which has been confirmed by most studies (14).

IVIg treatment is one of the elective treatment strategies in GBS and is superior to plasmapheresis in several centers, mainly because of its easy administration and wider availability. In several cases, plasmapheresis could shorten the time to recover the ability to walk independently (15). All the patients in our study received the IVIg treatment, and no related side effects were noted. Two patients who received IVIg and plasmapheresis showed a poor prognosis, which suggested that for some patients who were severely affected by GBS and had a slow response to initial IVIg therapy, combination with other potential therapeutic candidates was needed to limit the extent of nerve injury.

Besides motor weakness, up to two-thirds of patients with GBS are associated with autonomic dysfunction (16), and mortality can be approximately 7% in this patient population. One cohort study about autonomic dysfunction in childhood GBS had shown that the most common signs included hypertension and tachycardia, which usually occurred 9–15 days since symptom onset and were significantly correlated with the Hughes score (17). In our study, 13 (36.1%) children with GBS experienced hypertension and tachycardia. Furthermore, autonomic dysfunction was significantly more frequent in the slow-response group, suggesting that it was important to emphasize monitoring for cardiovascular disorders in the acute phase of GBS, especially for slow-response patients. Consistent with the results of our study, autonomic dysfunction in childhood GBS was often transient, and most children (approximately 80–90%) fully recovered upon discharge (18).

Approximately 15–24% of pediatric patients required mechanical ventilation owing to respiratory insufficiency (19). Several authors have reported that the risk factors associated with respiratory failure in GBS included an Erasmus GBS respiratory insufficiency score above five and facial and bulbar weakness (10). In our study, four (11.1%) patients who experienced mechanical ventilation showed a slow response to IVIg treatment and had a poor short-term prognosis. Therefore, this study suggests pursuing a better treatment option, such as the Zipper method of Hacettepe (which is a rigorous implementation of plasma exchange and IVIg in an interpenetrating manner) (20) and IVIg with other immune modulators, including complement inhibitors, for severe patients with mechanical ventilation.

GBS can be difficult to diagnose in children, mainly due to its atypical symptom presentation and the challenging neurological examination. An electrodiagnostic study can help support the GBS diagnosis to differentiate demyelinating and axonal variants and then correlate those findings to prognosis (21). Several retrospective analyses have indicated that AIDP was the most common underlying subtype, which was confirmed by our study as the ratios were 83.3% (30/36) and 16.7% (6/36) for AIDP and AMAN, respectively. In previous studies, patients with AIDP usually had a better prognosis than those with AMAN; however, no difference in the rate of response to IVIg treatment between them was noted. A-waves occur after F responses, and abundant A-waves are common in AIDP and are promising as a marker of demyelination (22, 23). Compared with non-A-waves, GBS with A-waves had poor clinical motor function and short-term prognosis but there was also no difference in the rate of response to IVIg treatment. All of the above studies had indicated that some factors associated with a good prognosis, such as AIDP subtype and GBS with non-A-waves, might not indicate a rapid response to treatment.

The limitations of our study were its retrospective nature and the relatively small sample size. We expect more prospective studies to enroll more childhood patients in the future, so as to better investigate the association between the rate of treatment response and short-or long-term prognosis in childhood Guillain-Barré Syndrome.

In conclusion, the rate of response to initial IVIg treatment is correlated with the short-term outcome of GBS in children, and the response time could be defined as 7 days. Patients whose response time was within 7 days had a good outcome. Moreover, the rate of treatment response had a predictive value for prognosis. The role of patients' initial responses to treatment could be significantly valuable in developing more effective and efficient treatment options.

## Data Availability Statement

The original contributions presented in the study are included in the article/supplementary material, further inquiries can be directed to the corresponding author.

## Ethics Statement

The studies involving human participants were reviewed and approved by the Ethics Committee of Children′s Hospital of Hebei Province. Written informed consent to participate in this study was provided by the participants' legal guardian/next of kin.

## Author Contributions

LZ and JX collected serum and CSF samples. JL acquired the electrophysiological data. WG and ZZ completed the statistical analysis. MJ designed the experiments, interpreted the results, and drafted the initial manuscript. CL reviewed the data and revised the manuscript. SS revised the initial draft and wrote the final manuscript. All authors contributed to the article and approved the submitted version.

## Funding

This work was supported by grants from the Medical Science Research Key Project Plan of Hebei Province in 2020 (20200223).

## Conflict of Interest

The authors declare that the research was conducted in the absence of any commercial or financial relationships that could be construed as a potential conflict of interest.

## Publisher's Note

All claims expressed in this article are solely those of the authors and do not necessarily represent those of their affiliated organizations, or those of the publisher, the editors and the reviewers. Any product that may be evaluated in this article, or claim that may be made by its manufacturer, is not guaranteed or endorsed by the publisher.

## Abbreviations

GBS: Guillain-Barré syndrome
IVIg: intravenous immunoglobulin
CSF: cerebrospinal fluid
AIDP: acute inflammatory demyelinating polyneuropathy
AMAN: acute motor axonal neuropathy.
