# Corrigendum: Association Between the Rate of Treatment Response and Short-Term Outcomes in Childhood Guillain-Barré Syndrome

**DOI:** 10.3389/fneur.2022.850357

**Published:** 2022-01-28

**Authors:** Mei Jin, Libo Zhao, Jing Liu, Weijin Geng, Ziwei Zhao, Chunzhen Li, Jingru Xue, Suzhen Sun

**Affiliations:** ^1^Department of Pediatrics, Hebei Medical University, Shijiazhuang, China; ^2^Department of Pediatric Neurology, Children's Hospital of Hebei Province, Shijiazhuang, China

**Keywords:** Guillain-Barré syndrome, children, treatment response rate, Hughes Functional Grading Scale, short-term outcomes

In the published article, there was an error regarding the affiliations for Suzhen Sun. As well as having affiliation “2”, they should also have “Department of Pediatrics, Hebei Medical University, Shijiazhuang, China.”

The authors apologize for this error and state that this does not change the scientific conclusions of the article in any way. The original article has been updated.

## Publisher's Note

All claims expressed in this article are solely those of the authors and do not necessarily represent those of their affiliated organizations, or those of the publisher, the editors and the reviewers. Any product that may be evaluated in this article, or claim that may be made by its manufacturer, is not guaranteed or endorsed by the publisher.

